# Exploration of molecular biomarkers in ankylosing spondylitis transcriptomics

**DOI:** 10.3389/fimmu.2024.1480492

**Published:** 2024-12-20

**Authors:** Yuanpiao Ni, Linrui Zhong, Yanhui Li, Zeng Zhang, Bin Ming, Yufeng Qing, Quanbo Zhang

**Affiliations:** ^1^ Research Center of Hyperuricemia and Gout, Affiliated Hospital of North Sichuan Medical College, Nanchong, Sichuan, China; ^2^ Department of Rheumatology and Immunology, Affiliated Hospital of North Sichuan Medical College, Nanchong, Sichuan, China; ^3^ Department of Geriatrics, Affiliated Hospital of North Sichuan Medical College, Nanchong, China

**Keywords:** ankylosing spondylitis, weighted gene co-expression network analysis, differential gene expression, immune infiltration analysis, ferroptosis, transcriptomics

## Abstract

**Background:**

Inflammation of the spine and sacroiliac joints is a hallmark of the chronic, progressive inflammatory illness known as ankylosing spondylitis (AS). The insidious onset and non-specific early symptoms of AS often lead to delays in diagnosis and treatment, which may result in the onset of disability. It is therefore imperative to identify new biomarkers.

**Methods:**

In this study, datasets GSE73754 and GSE25101 were derived from the Gene Expression Omnibus (GEO). Key genes were identified through differential expression analysis and weighted gene co-expression network analysis (WGCNA). A model was then established using LASSO regression, and then it was subjected to the receiver operating characteristic (ROC) curve analysis for evaluation of the diagnostic accuracy of the genes. Subsequently, immune infiltration analysis was conducted to demonstrate the immune infiltration status of the samples and the correlation between key genes and immune infiltration. Finally, the expression levels of key genes in peripheral blood mononuclear cells (PBMCs) and their correlation with clinical indicators were validated via RT-qPCR assay.

**Results:**

Through WGCNA and differential expression analysis, 6 genes were identified. Ultimately, five key genes (ACSL1, SLC40A1, GZMM, TRIB1, XBP1) were determined using LASSO regression. The area under the ROC curve (AUC) for these genes was greater than 0.7, indicating favorable diagnostic performance. Immune infiltration analysis showed that AS was associated with infiltration levels of various immune cells. RT-qPCR validated that the expression of ACSL1, SLC40A1, GZMM, and XBP1 was consistent with the predictive model, whereas TRIB1 expression was contrary to the predictive model. Clinical correlation analysis of key genes revealed that ACSL1 was positively linked to hsCRP levels, GZMM was negatively linked to, hsCRP levels, and neutrophil absolute values, SLC40A1 was positively linked to ESR, hsCRP levels and neutrophil absolute values, and XBP1 was negatively linked to ESR, hsCRP levels, and neutrophil absolute values.

**Conclusion:**

This study identified key genes that may reveal a potential association between AS and ferroptosis, demonstrating high diagnostic value. Furthermore, the expression levels of these genes in peripheral blood mononuclear cells (PBMCs) are strongly correlated with disease activity. These findings not only suggest potential biomarkers for the diagnosis of AS but also offer important references for exploring new therapeutic targets, highlighting their substantial clinical applicability.

## 1 Introduction

Ankylosing spondylitis (AS) is a chronic, progressive inflammatory disease involving the spine and sacroiliac joints (1, 2). The prevalence of AS varies significantly across different regions, ranging from 0.74% in Africa to 3.19% in North America (3). In addition to inflaming the sacroiliac joints and spine, AS can cause extra-articular symptoms such as anterior psoriasis, uveitis, or inflammatory bowel disease (4). In severe stages of the disease, persistent inflammation can cause fibrosis and calcification of spinal joints, restricting joint mobility and potentially leading to joint fusion, which may ultimately result in disability (5). Due to the insidious onset and subtle early symptoms of AS, its diagnosis is often delayed. For instance, in the United States, the average time from symptom onset to referral is approximately one year, whereas in Western Europe and other regions, this time can exceed three years, frequently leading to missed treatment opportunities and delayed therapy (6).

Although the exact cause of AS is still unknown, environmental and genetic factors such as autophagy, inflammatory cytokines, certain bacterial infections, and macrophage activation are thought to have a role in its pathogenesis (7). Due to a strong familial predisposition of AS, early research highlighted the significance of genetic factors in its pathogenesis. However, as our understanding of the disease has deepened, it has become evident that the currently used biomarkers, such as HLA-B27 status, C-reactive protein (CRP), and erythrocyte sedimentation rate (ESR), provide only moderate diagnostic and prognostic utility. There is a pressing need for improved biomarkers in AS to facilitate early diagnosis, improve prediction of therapeutic responses, and facilitate the assessment of long-term outcomes in AS. Recent advancements in transcriptomics technologies and statistical methodologies offer promising opportunities to identify and develop more informative biomarkers for such clinical applications (8, 9).

RNA sequencing (RNA-seq) has become a novel high-throughput sequencing technique in recent decades. It is capable of recognizing abnormally spliced genes, detecting allele-specific expression, and identifying differentially expressed genes (DEGs) (10). Bioinformatics analysis has been utilized to elucidate abnormal biological processes underlying disease pathogenesis and can leverage sequencing data to assess an organism’s genome, transcriptome, and proteome information (11). To date, several studies have identified DEGs implicated in the pathogenesis of AS using microarray and RNA-seq techniques. Peripheral blood is widely recognized as a promising resource for identifying transcriptomic biomarkers (12). Notably, peripheral blood biomarkers have achieved significant success in predicting tumor onset and progression, such as in lung and breast cancers, as well as in the detection and drug development of Alzheimer’s disease (13–15). Nevertheless, the DEG levels in the peripheral blood of AS patients have yet to be fully elucidated, and the aforementioned molecular mechanisms remain to be further validated.

In this study, gene microarray expression data from GSE73754 and GSE25101 were obtained from the GEO database. Using bioinformatics analysis, DEGs in the serum of patients with AS and normal controls were identified. Gene Ontology (GO), Kyoto Encyclopedia of Genes and Genomes (KEGG), and Gene Set Enrichment Analysis (GSEA) were performed to explore their functions and pathways. Then, key genes were identified, and further receiver operating characteristic (ROC) analysis of these key genes was conducted. This study identified a strong association between the key genes and ferroptosis, a newly recognized form of programmed cell death with a critical role in various inflammatory diseases. In patients with ankylosing spondylitis (AS), abnormalities in iron metabolism and oxidative stress are hallmark pathological features, suggesting that ferroptosis may significantly contribute to the development and progression of AS (16). Furthermore, through immune infiltration analysis, this study explored the disease microenvironment of AS, uncovering the potential involvement of immune cells in its pathogenesis. As AS is a chronic inflammatory disease characterized by immune abnormalities, the findings on immune infiltration offer deeper insights into its immunopathological mechanisms (2). Finally, the expression levels of key genes and their correlation with various clinical indicators were validated by real-time quantitative PCR. In addition to offering fresh information on the pathological mechanisms of AS, the study suggested new potential biomarkers and targets for AS diagnosis and treatment.

## 2 Materials and methods

### 2.1 Data collection

Gene expression data GSE73754 and GSE25101 were derived from the GEO database (17, 18). Based on the GPL10558 platform, the GSE73754 dataset included 72 samples, of which 52 were peripheral blood samples from patients with AS while 20 were from healthy controls. Based on the GPL6947 platform, the GSE25101 dataset included 32 samples, comprising peripheral blood samples from 16 patients with AS and 16 healthy controls.

### 2.2 Differential expression analysis and enrichment analysis

In the GSE25101 and GSE73754 datasets, DEGs were screened. DEGs were identified using the Limma R package, with a significance threshold defined when the *P*-value was less than 0.05 and the FoldChange was greater than 1.2 (19). The R packages “ggplot2” and “pheatmap” were utilized to visually present the DEGs via volcano plots and heatmaps (19). GO and KEGG functional enrichment analyses were executed via the “clusterProfiler” and “enrichplot” R packages. Additionally, GSEA was conducted based on GSE73754 using the “clusterProfiler” and “ReactomePA” R packages to identify relevant enriched signaling pathways (20, 21).

### 2.3 Weighted gene co-expression network construction and module analysis

Each gene’s median absolute deviation (MAD) was determined, and the top 50% of genes were chosen based on their MAD values. To examine the connection between co-expressed genes and phenotypes, a gene co-expression network was built (22). Gene comparison was done via average linkage methods and Pearson correlation matrices. By utilizing the weighted adjacency matrix and the soft-thresholding parameter β, a scale-free co-expression network was established. The adjacency matrix was raised to the power of 14 to convert into a topological overlap matrix (TOM), which was used to gauge the network connectivity of genes. With the average linkage hierarchical clustering and TOM-based dissimilarity measures, the correlation among modules was identified, with the minimum gene module size set to 10.

### 2.4 Core gene selection and logistic regression model construction

The intersection of co-expressed DEGs from GSE73754 and GSE25101 datasets with WGCNA module genes identified 6 genes. LASSO regression was then adopted to simplify the model, identifying 5 key genes, which were utilized to establish a diagnostic model for AS (23). The diagnostic performance of key genes and the logistic regression model were evaluated using the R package “ROCR”. Subsequently, a nomogram for predicting AS risk based on characteristic genes was constructed using the R package “rms”, and its predictive efficacy was estimated through calibration curves.

### 2.5 Immune analysis algorithm

Based on the expression levels of genes relevant to immune cells, the ssGSEA algorithm was adopted to determine the infiltration levels of different immune cells. An immune cell composition matrix for analysis was created by integrating the output data for 28 different categories of immune cells. The correlation between core biomarkers and immune infiltrating cell expression was analyzed using non-parametric Spearman correlation. The “corrplot” R package was then employed to draw correlation heatmaps.

### 2.6 Study procedure

From Mar. 2024 to Jun. 2024, 24 drug-naive patients with AS meeting the modified New York diagnostic criteria (1984) were selected from the Affiliated Hospital of North Sichuan Medical College, and blood samples were collected from 24 healthy male volunteers (healthy control group, HC group) (24). All participants had no history of cardiovascular disease, diabetes mellitus, hepatitis, malignancies, or other autoimmune and inflammatory diseases. The study was approved by the Affiliated Hospital of North Sichuan Medical College’s Ethics Committee, with informed consent obtained from all participants (Approval No.: 2024ER268-1). 4 mL of heparin-anticoagulated peripheral venous blood was used for the isolation of peripheral blood mononuclear cells (PBMCs). The Trizol technique was utilized to extract total RNA. Gene primers were designed as per the gene sequences of ACSL1, SLC40A1, GZMM, TRIB1, and XBP1 in PubMed Gene and synthesized by Sangon Biotech (Shanghai) Co., Ltd. (**Table 1**).

**Table 1:** Primer sequences of mRNA for qRT-PCR.

	Primer sequence, 5’–3’ Forward	Reverse
Gene (hum)		
β-Actin	GAGCTACGAGCTGCCTGACG	GTAGTTTCGTGGATGCCACAG
ACSL1	CTTATGGGCTTCGGAGCTTTT	CAAGTAGTGCGGATCTTCGTG
GZMM	ACACCCGCATGTGTAACAACA	GGAGGCTTGAAGATGTCAGTG
SLC40A1	CTACTTGGGGAGATCGGATGT	CTGGGCCACTTTAAGTCTAGC
TRIB1	GCTGCAAGGTGTTTCCCATTA	TCCCCAAAGTCCTTCTCAAAGA
XBP1	CCCTCCAGAACATCTCCCCAT	ACATGACTGGGTCCAAGTTGT

### 2.7 Statistical analysis

All bioinformatics statistical analyses and visualizations were performed using R (version 4.3.2). Values from the experiment were reported as mean ± standard deviation (SD). In addition, to verify the data normality, the Shapiro-Wilk test was adopted. The t-test or the Mann-Whitney U test was adopted for the analysis based upon whether the data set exhibited a normal distribution, while Pearson or Spearman tests were utilized for correlation analysis. All experimental statistical analyses were conducted using SPSS v27 or GraphPad Prism (v10.2.3). At least three independent replications of each experiment were conducted. *P* < 0.05 was used to define significance.

## 3 Results

### 3.1 DEG identification in ankylosing spondylitis

**Figure 1:**
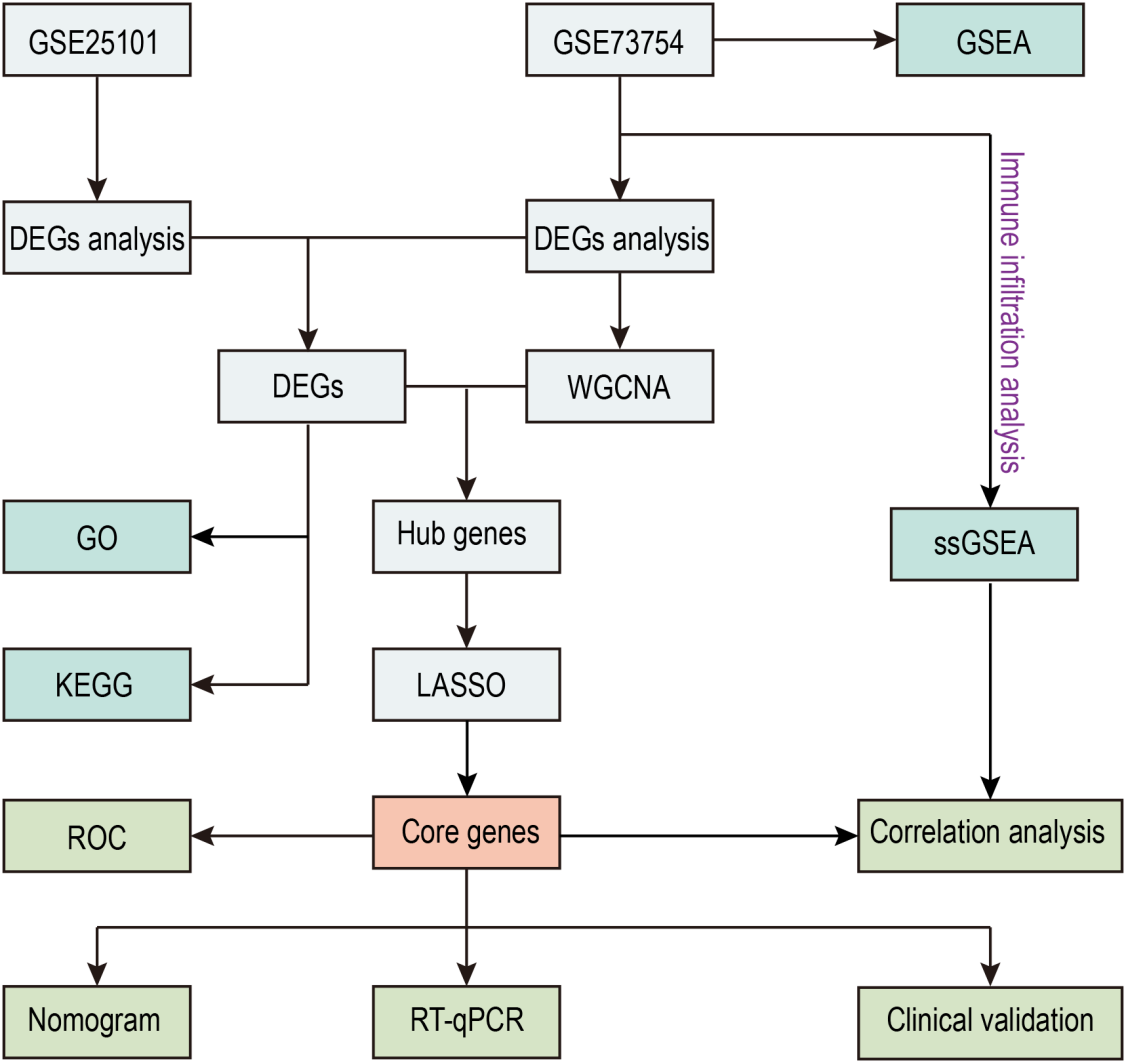
Flow diagram of the study.

**Figure 2:**
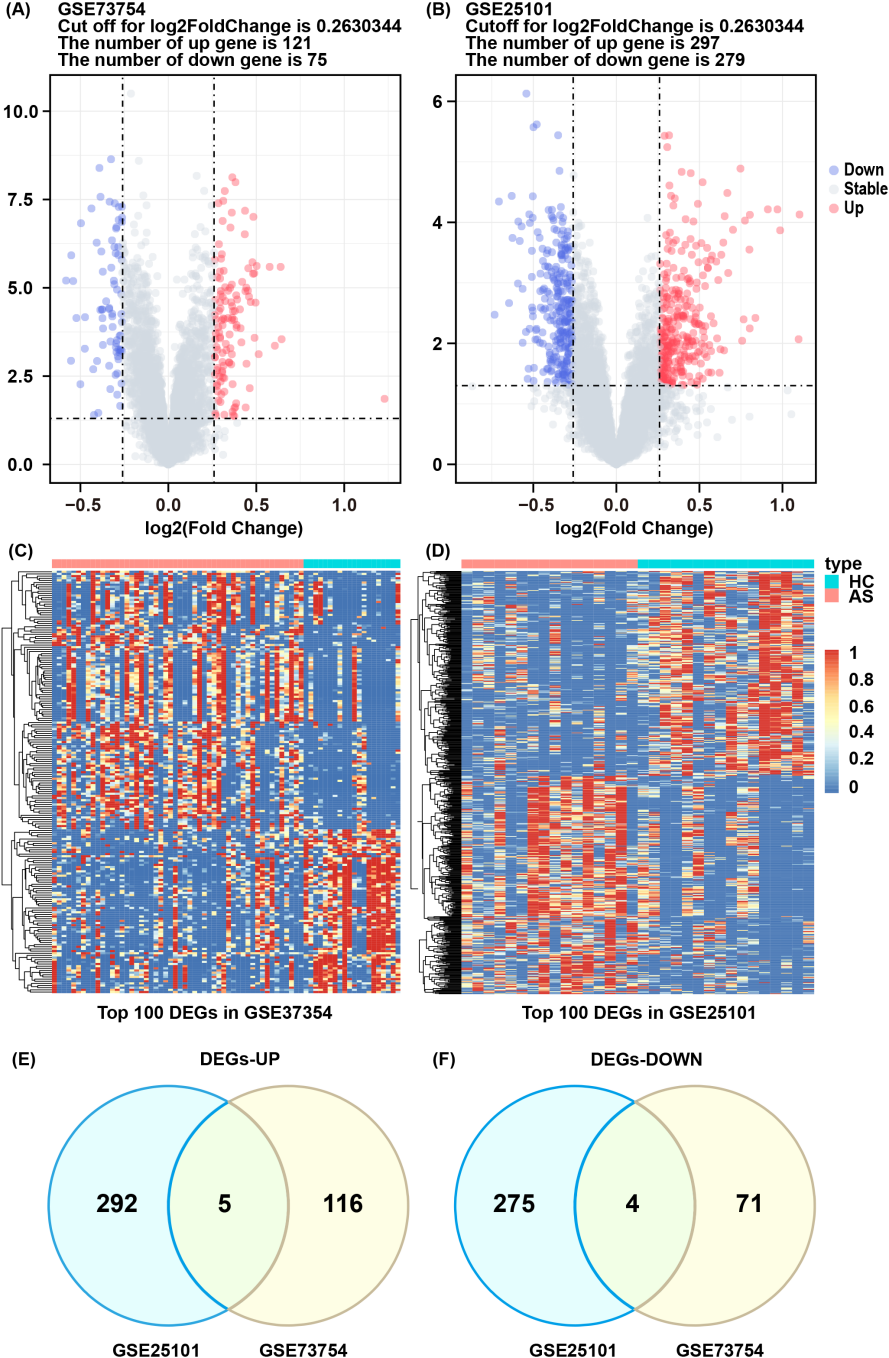
DEGs and enrichment analysis of AS. **(A, B)** Volcano plot of DEGs between AS and HC groups in GSE73754 and GSE25101. **(C, D)** Heatmap of top 100 DEGs in GSE73754 and GSE25101. **(E)** Commonly upregulated differentially expressed genes. **(F)** Commonly downregulated differentially expressed genes.

The flowchart of this study is illustrated in **Figure 1**. We investigated high-throughput sequencing data from the GSE73754 and GSE25101 databases, pertaining to patients with AS and healthy controls. A total of 196 DEGs were successfully identified from the GSE73754 dataset, including 121 upregulated genes and 75 downregulated genes. From the GSE25101 dataset, 576 DEGs were identified, comprising 297 upregulated genes and 279 downregulated genes. These DEGs were visualized using volcano plots (**Figures 2A, B**), and the top 100 DEGs were presented in heatmaps (**Figures 2C, D**). Based on the differential gene expression trends from these two datasets, 9 co-expressed DEGs were obtained, with 5 upregulated genes (**Figure 2E**) and 4 downregulated genes (**Figure 2F**).

### 3.2 Potential function and pathway analysis

**Figure 3:**
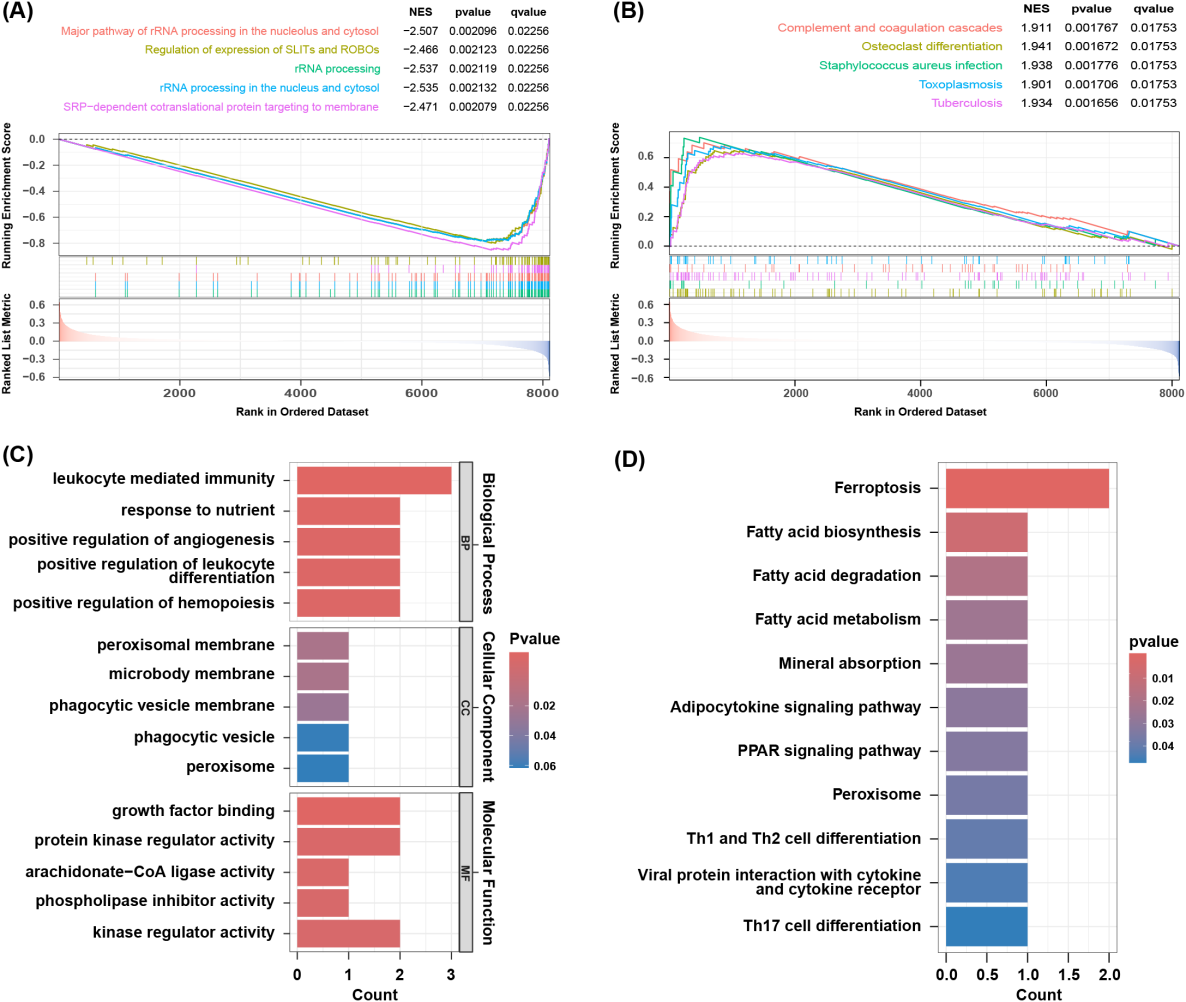
Result of functional enrichment analysis. **(A)** The significant GSEA sets in GO. **(B)** The significant GSEA sets in KEGG pathways. **(C)** GO analysis of co-DEGs. **(D)** KEGG pathways of co-DEGs.

GSEA analysis of the GSE73754 dataset showed significant downregulation of rRNA processing and translation processes (**Figure 3A**), indicating that these key biological processes were potentially suppressed under the studied conditions. Additionally, osteoclast differentiation-, *Streptococcus* infection-, and tuberculosis-related genes were significantly upregulated, while oxidative phosphorylation process-related genes were downregulated (**Figure 3B**), suggesting changes in immune response and metabolic activity in AS. GO and KEGG enrichment analyses were performed on the 9 co-expressed DEGs obtained by integrating the GSE73754 and GSE25101 datasets. GO analysis identified 188 significantly different GO terms, including 162 biological processes, 3 cellular components, as well as 23 molecular functions. These genes were enriched in key biological processes such as leukocyte-mediated immunity and positive regulation of angiogenesis. Regarding the cellular component, the peroxisomal membrane and phagocytic vesicle membrane were highlighted. In terms of the molecular function, growth factor binding and protein kinase regulator activity pathways were enriched (**Figure 3C**). These genes are mainly involved in immune response, cytotoxicity, and regulatory processes, indicating a close association between AS and abnormal immune responses and cellular regulatory mechanisms. KEGG analysis unraveled that 11 pathways were enriched, including ferroptosis, fatty acid metabolism, mineral absorption, as well as Th1, Th2, and Th17 cell differentiation (**Figure 3D**). The enrichment of these pathways indicated substantial alterations in immune response, metabolic activity, and cell death mechanisms in AS, offering crucial insights into the underlying biological processes.

### 3.3 Construction of weighted gene co-expression network

**Figure 4:**
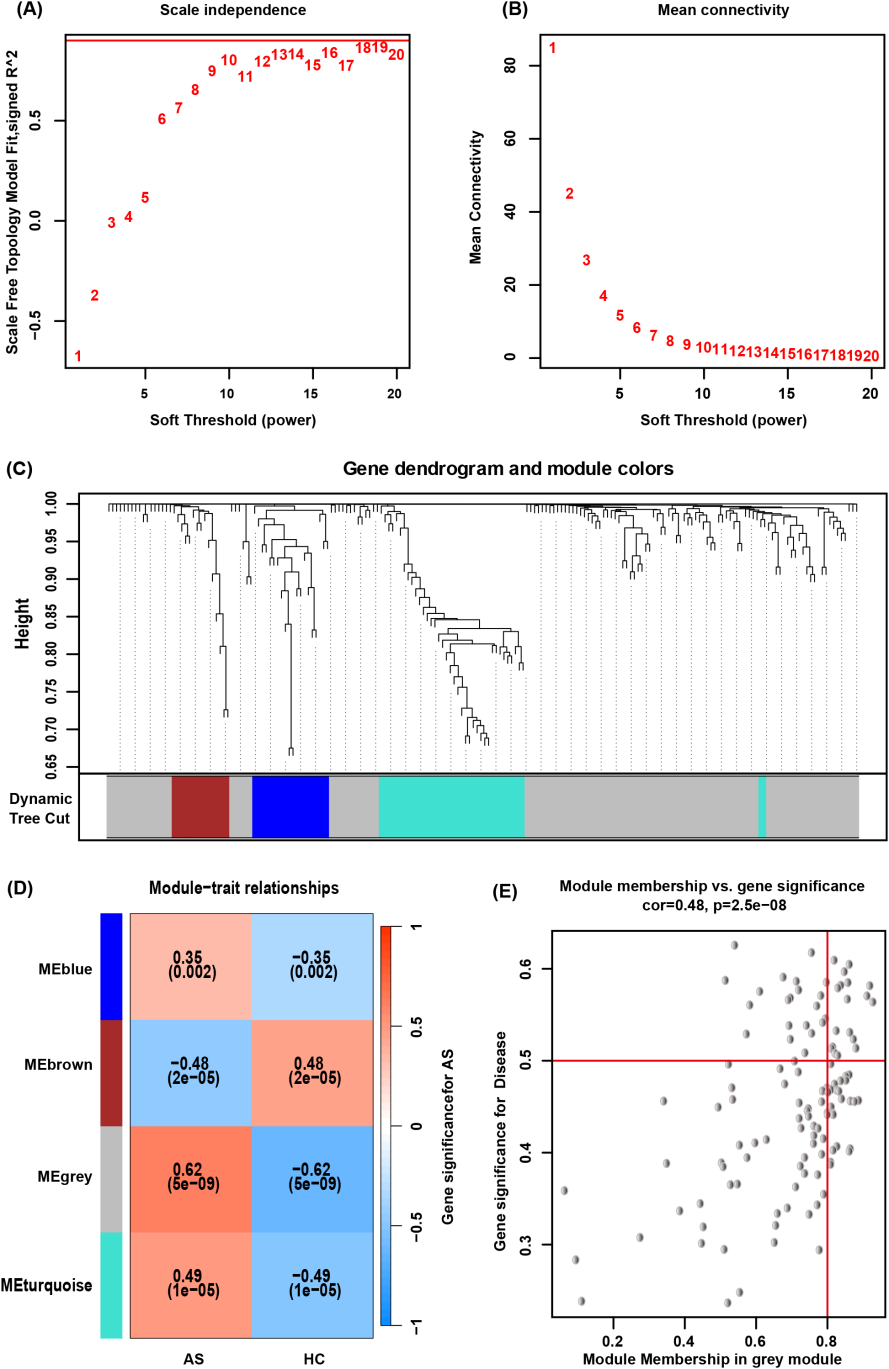
WGCNA. **(A, B)** Determination of an optimal soft-thresholding power β by calculating the scale-free topology model fit and mean connectivity. **(C)** The cluster dendrogram of mRNAs in GSE73754, revealing different mRNA co-expression modules marked with colors. **(D)** The heatmap for module-traits relationships, showing the correlation of different modules with AS or HC. **(E)** Relationship between Module Membership and Gene Significance for AS.

By WGCNA, co-expressed gene clusters with differential expression in the GSE73754 dataset were identified, and the relationship between combined modules and disease traits was calculated (**Figure 4C**). The soft-threshold power was set to 15 (**Figures 4A, B**), and 4 modules were identified (**Figure 4D**). The grey module showed the most robust positive correlation with the occurrence of AS (r = 0.48), and 121 genes from the grey module were screened (**Figure 4E**).

### 3.4 Validation of the diagnostic model based on key genes

**Figure 5:**
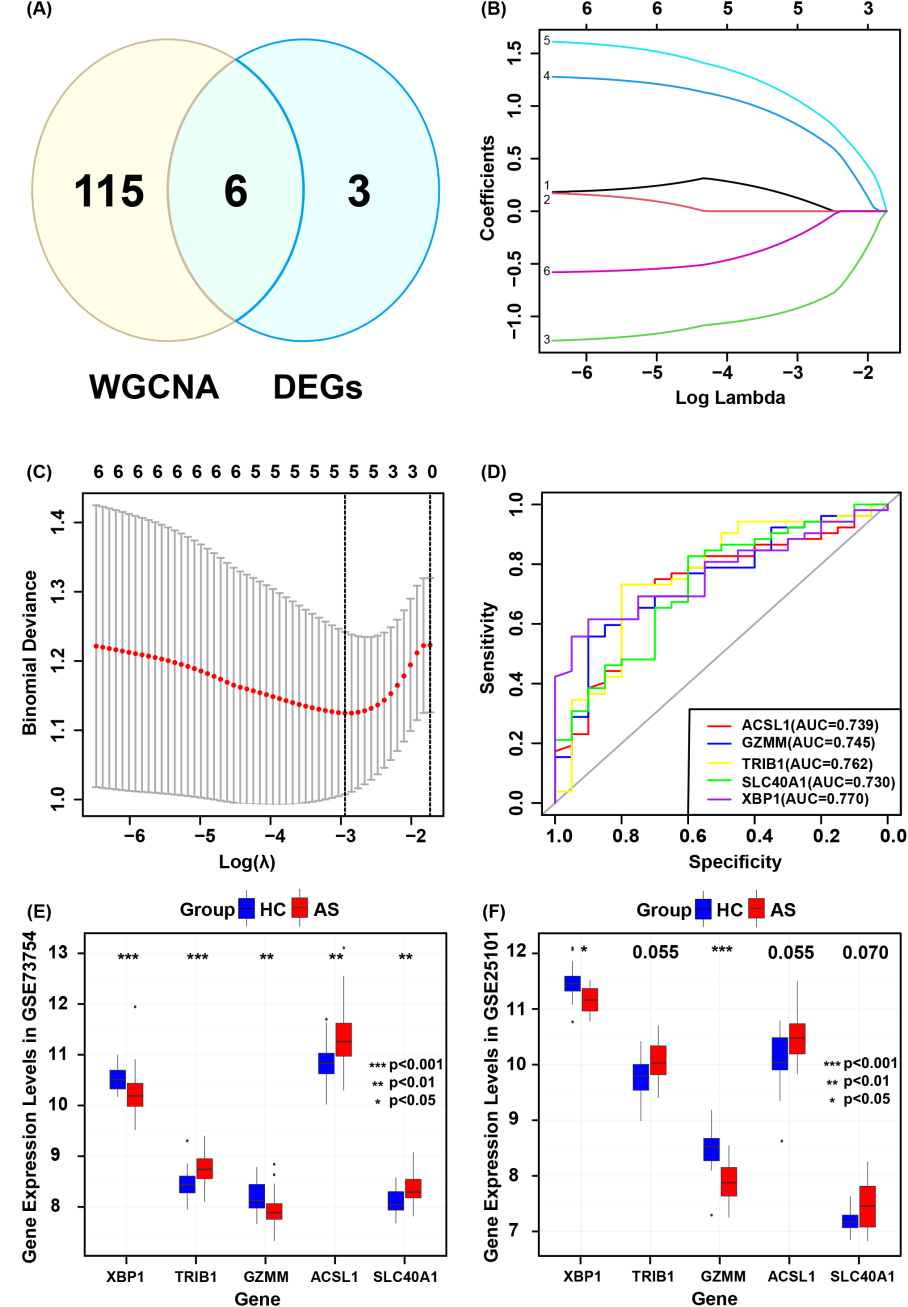
Validation of core genes diagnostic models. **(A)** Intersection of genes identified by WGCNA and co-expressed genes. **(B)** Selection of optimal parameters (lambda) in the LASSO model. **(C)** Five core genes identified by the optimal lambda. **(D)** ROC curves for each core gene. **(E)** Core gene expression levels in GSE73754. **(F)** Core gene expression levels in GSE25101.

**Figure 6:**
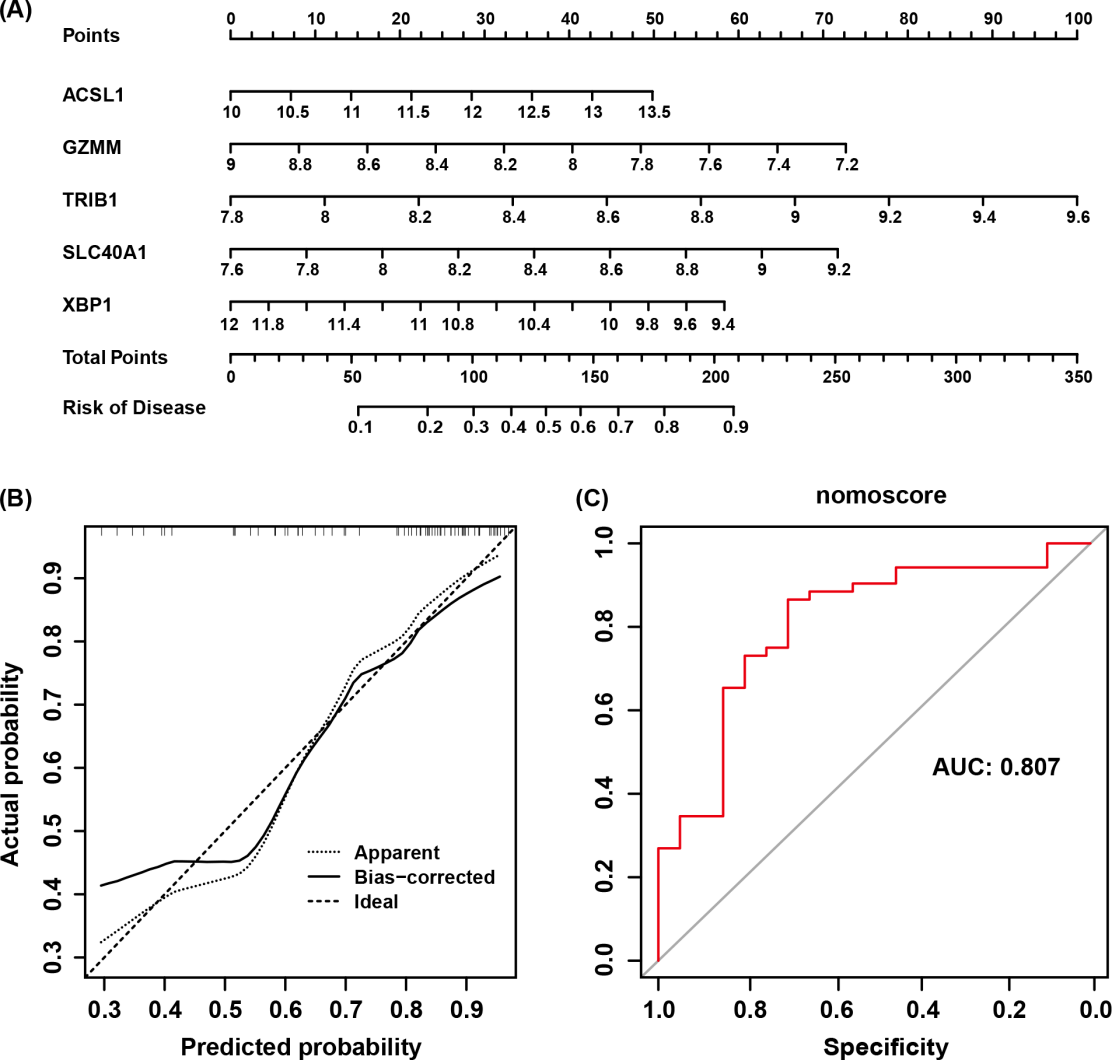
**(A)** Nomogram model of AS. **(B)** Calibration curve of the nomogram testing the predictive performance of the model. **(C)** AUC of the diagnostic model based on core genes.

The intersection of the 9 co-expressed DEGs from the GSE73754 and GSE25101 datasets with the 121 grey module genes identified by WGCNA resulted in 6 core genes (**Figure 5A**). Using LASSO regression to simplify the model (**Figures 5B, C**), 5 key genes (ACSL1, SLC40A1, GZMM, TRIB1, and XBP1) were identified. Box plots were then adopted to show the expression trends of these 5 key genes in the GSE73754 and GSE25101 datasets (**Figures 5E, F**). Subsequently, ROC analysis was made to evaluate the potential of these 5 key genes as diagnostic biomarkers for AS (**Figure 5D**). All key genes had an AUC greater than 0.7, indicating favorable clinical diagnostic performance. A nomogram predicting AS risk based on these 5 key genes was constructed (**Figure 6A**), with each gene corresponding to a scoring standard. The calibration curve attested to the favorable predictive performance of the model (**Figure 6B**). Additionally, the ROC curve analysis showed that the overall AUC of the model was 0.807 (**Figure 6C**), indicating that the core genes have high diagnostic performance.

### 3.5 Correlation analysis concerning immune cell infiltration and key genes

**Figure 7:**
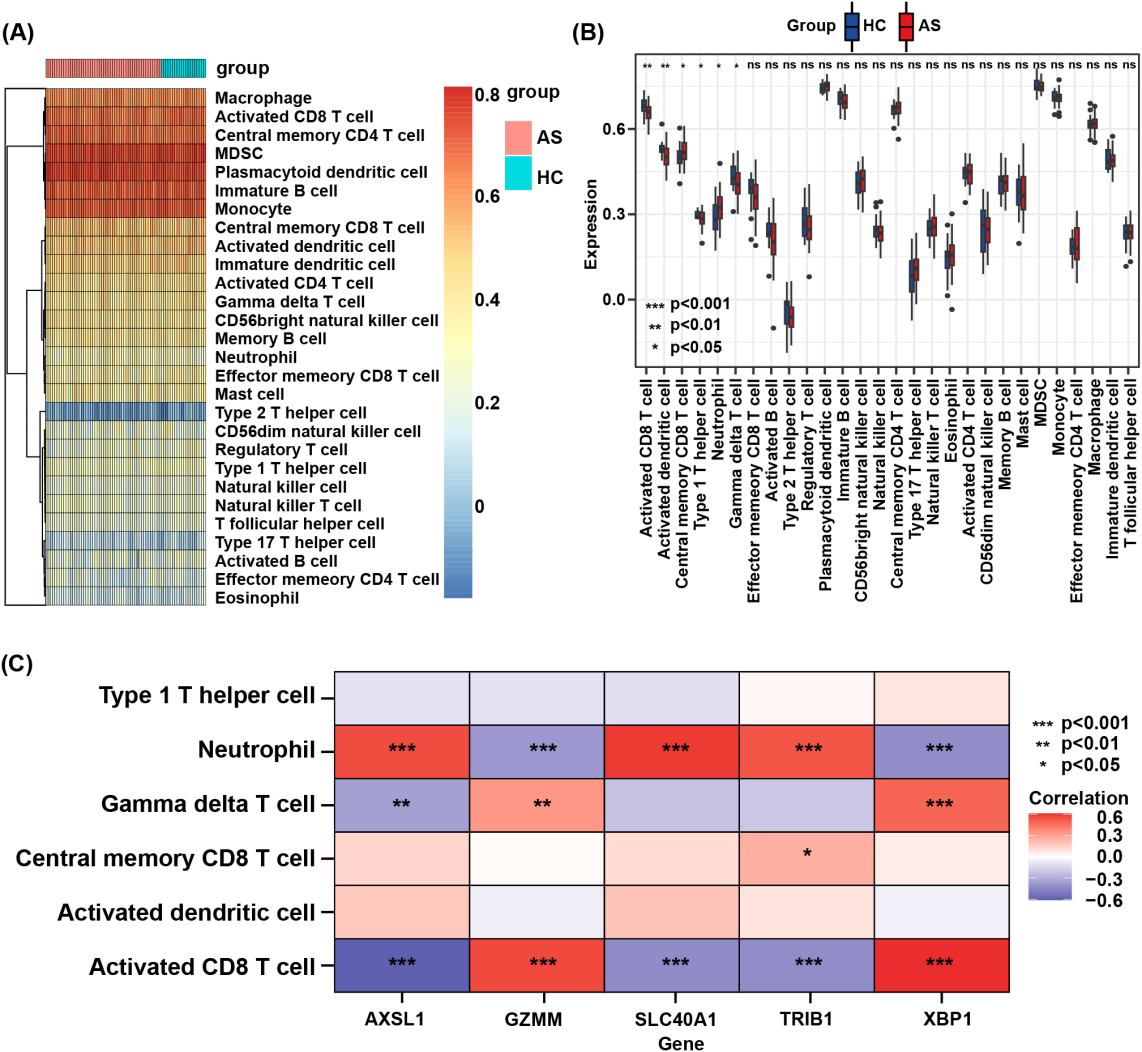
**(A)** Heatmap of the proportions of 28 immune cells in the AS and HC groups. **(B)** Boxplot of the immune cell proportions in AS and HC groups. “ns” means “not significant”. **(C)** Correlation analysis between core genes and differential immune cell subsets.

An analysis of the infiltration of 28 immune cell subtypes between the AS group and the control group revealed that six immune cell subsets showed statistically significant differences (**Figure 7A**). In the AS group, central memory CD8 T cells and neutrophils were increased, while activated CD8 T cells, activated dendritic cells, type 1 helper T cells, and γδT cells were decreased (**Figure 7B**). Furthermore, the correlation analysis between key genes and the aforementioned differential immune cell subsets showed that ACSL1, SLC40A1, and TRIB1 were positively linked to neutrophil infiltration, whereas GZMM and XBP1 were negatively linked to neutrophil infiltration. GZMM and XBP1 were positively linked to γδT cell infiltration, while ACSL1 was negatively linked to γδT cell infiltration. TRIB1 was positively linked to central memory CD8 T cell infiltration. GZMM and XBP1 were positively linked to activated CD8 T cell infiltration, while ACSL1, SLC40A1, and TRIB1 were negatively linked to activated CD8 T cell infiltration (**Figure 7C**).

### 3.6 RT-qPCR expression and clinical correlation

**Figure 8:**
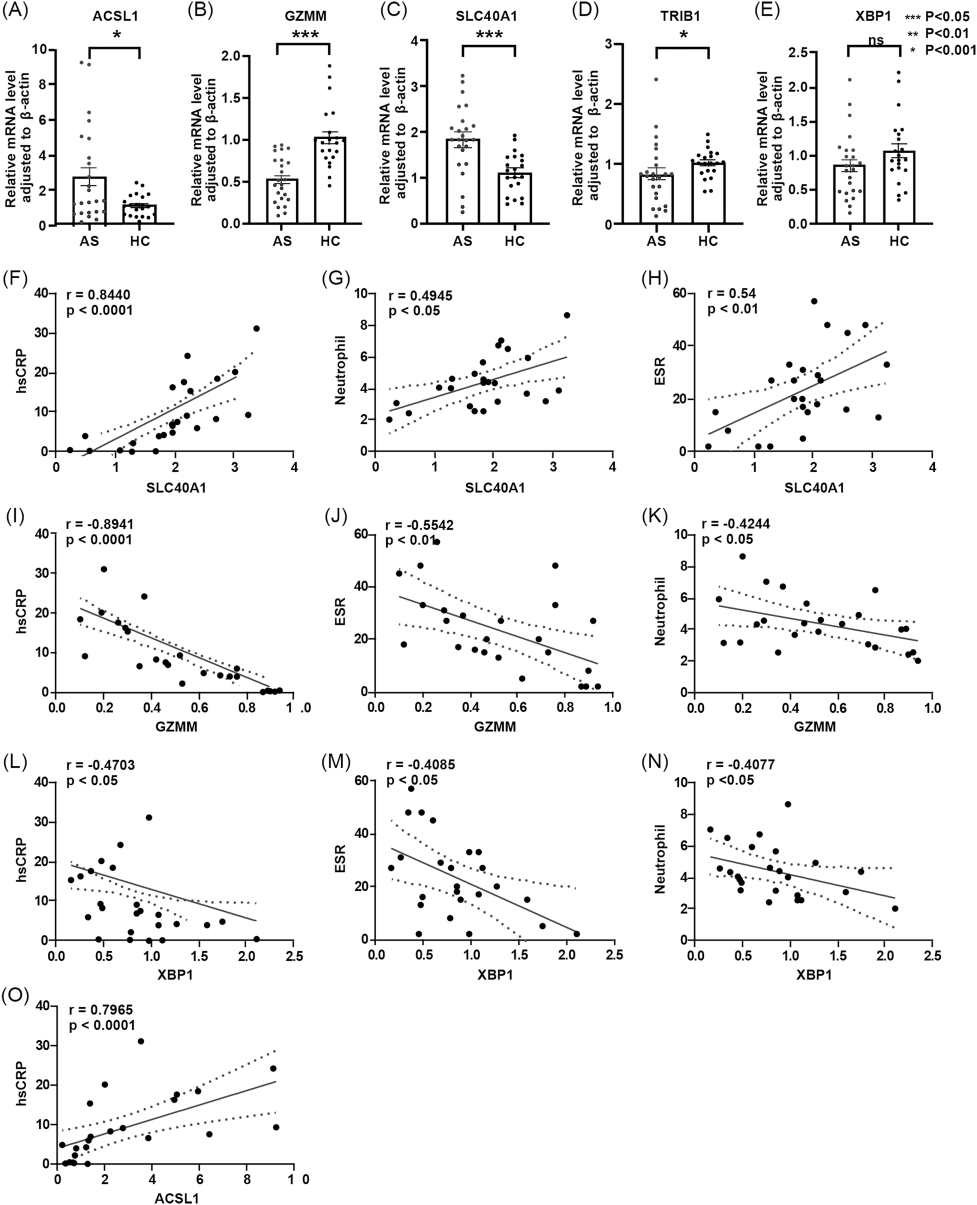
**(A-E)** Differences in relative expression levels of five core genes between AS group and HC group. – **(A)** ACSL1 – **(B)** GZMM – **(C)** SLC40A1 – **(D)** TRIB1 – **(E)** XBP1. **(F-O)** Correlation analysis between core genes and clinical data. – **(F)** Correlation between SLC40A1 and hsCRP- **(G)** Correlation between SLC40A1 and neutrophil count – **(H)** Correlation between SLC40A1 and ESR – **(I)** Correlation between GZMM and hsCRP – **(J)** Correlation between GZMM and ESR – **(K)** Correlation between GZMM and neutrophil count – **(L)** Correlation between XBP1 and hsCRP – **(M)** Correlation between XBP1 and ESR – **(N)** Correlation between XBP1 and neutrophil count – **(O)** Correlation between ACSL1 and hsCRP.

To validate the role of key genes, an RT-qPCR assay of the mRNA expression levels of the five key genes in PBMCs from AS patients and healthy individuals was performed (**Figures 8A–E**). The results showed that, compared to healthy controls, the expression level of ACSL1 and SLC40A1 was upregulated in AS, while GZMM and XBP1 expression was downregulated, consistent with the expression trends predicted by the model. However, TRIB1 expression was downregulated, contrary to predicted expression by the model (**Figures 5E, F**). Correlation analysis between the five key genes and clinical indicators (including blood analysis, hsCRP, ESR, HLA-B27, gender, and age) (**Table 2**) showed that ACSL1 was positively linked to patient hsCRP levels (r = 0.7965, *P* < 0.0001). GZMM was negatively linked to patient ESR levels (r = -0.5542, *P* < 0.05), hsCRP levels (r = -0.8941, *P* < 0.05), and neutrophil count (r = -0.4244, *P* < 0.05). SLC40A1 was positively linked to patient ESR levels (r = 0.54, *P* < 0.01), hsCRP levels (r = 0.8440, *P* < 0.0001), and neutrophil count (r = 0.4945, *P* < 0.05). XBP1 was negatively linked to patient ESR levels (r = -0.4085, *P* < 0.05), hsCRP levels (r = -0.4703, *P* < 0.05), and neutrophil count (r = -0.4077, *P* < 0.05) (**Figures 8F–O**).

**Table 2:** General information of AS patients and healthy control donors.

	AS group (n=24)	HC group (n=24)
Gender(male/female)	18/6	17/7
Age (years)	34.6 ± 6.9	36.5 ± 8.2
Positive rate of HLA–B27	91.67%	N/A
ESR, mm/hour	23.25 ± 15.41	N/A
hsCRP, mg/L	9.06 ± 8.45	N/A
ANC,109/L	4.38 ± 1.66	N/A

“N/A” means “Not Applicable”.

## 4 Discussion

This study identified five key genes (ACSL1, SLC40A1, GZMM, TRIB1, and XBP1) as potential biomarkers for AS by analyzing the GSE73754 and GSE25101 datasets from the GEO database. The mRNA expression levels of these key genes in PBMCs from AS patients and healthy individuals were validated using RT-qPCR assay. Furthermore, these genes may be linked to disease activity and may be useful in the diagnosis of AS, according to the correlation analysis between the key gene expression and clinical data.

AS typically leads to calcification and bone formation, accompanied by destructive bone lesions. New bone formation within the axial skeleton is a characteristic of post-inflammatory AS (25). Innate cytokines, specifically the interleukin-23/17 axis, have been shown in recent research to have a critical role in the pathogenesis of AS (26, 27). Clinically, anti-IL-17 therapy has been proven effective in improving bone destruction in AS, but some patients do not respond to IL-17 treatment (28). Our study found that the IL-17 pathway was also enriched in the co-expressed trend genes identified, consistent with current research. Additionally, KEGG analysis revealed that were adipocytokine signaling pathway, mineral absorption pathway, PPAR signaling pathway, and Th1 and Th2 cell differentiation pathways were enriched, all of which have been reported to have significant value in bone metabolism research (29–32). The study also identified several genes related to bone metabolism and osteocyte function. GO analysis revealed multiple biological processes related to osteocyte differentiation and bone metabolism, such as bone mineralization, osteoblast differentiation, and osteoclast differentiation. GSEA uncovered key pathways, including osteoclast differentiation, suggesting the active role of osteoclasts in AS. Additionally, GSEA revealed metabolic pathways such as oxidative phosphorylation. These pathways are closely related to osteoclast function and bone metabolism.

Among the core genes in this study, ACSL1 is a key enzyme in fatty acid metabolism, mainly responsible for converting long-chain fatty acids into acyl-CoA, a prerequisite for fatty acids to participate in metabolic pathways such as β-oxidation and lipid synthesis (33). Studies have shown that lipid molecules containing 18:3 chains significantly decline in cells lacking ACSL1, while the presence of ACSL1 increases the synthesis and accumulation of these lipids, which may promote ferroptosis through oxidation (34–36). The experiment results demonstrated that ACSL1 was upregulated in AS patients and exhibited a positive correlation with hsCRP levels. This suggested that ACSL1 may promote ferroptosis through fatty acid metabolism, thereby enhancing the inflammatory response in AS. SLC40A1 is an iron transporter protein responsible for transporting intracellular iron to the extracellular space, playing an important role in iron metabolism. SLC40A1 influences the iron content and function of immune cells such as dendritic cells by regulating iron export, thereby modulating immune responses (37, 38). According to the findings of our investigation, AS patients had upregulated SLC40A1, which positively linked to both hsCRP and ESR. This finding suggests that it may indirectly enhance the inflammatory response in AS by affecting the iron content of immune cells like dendritic cells. As a serine protease belonging to the granzyme family, GZMM is mostly released by cytotoxic lymphocytes, including CD8^+^ T cells and natural killer cells. It plays a role in immune defense and target cell lysis (39, 40). In this study, GZMM was downregulated in patients with AS and negatively linked to multiple immune cell infiltrations, suggesting that GZMM may inhibit inflammation by modulating the activity of neutrophils and γδT cells. Notwithstanding its lack of catalytic activity, the pseudokinase TRIB1 is crucial in cell signaling and gene expression regulation. It has an impact on various cellular processes, such as metabolism, inflammation, and cell differentiation (41). In this study, the RT-qPCR expression of TRIB1 in AS patients contradicted the predictive model, which may be attributable to various factors. The model’s prediction of elevated TRIB1 expression might reflect its role in diverse and complex cellular signaling pathways. In contrast, the lower RT-qPCR expression may result from changes in the proportion of specific cell subsets within PBMCs of AS patients, potentially affecting overall expression levels. Furthermore, TRIB1 expression could be dynamically regulated by microenvironmental factors, such as inflammatory cytokines and oxidative stress, contributing to the observed discrepancies between the model and experimental data. Moreover, the predictive model, which relies on big data statistical analysis, may be influenced by sample heterogeneity and data normalization methods. In comparison, RT-qPCR results depend on experimental conditions (e.g., RNA quality, primer design), with these technical differences possibly accounting for the observed inconsistencies. In this study, TRIB1 was downregulated in AS patients and positively correlated with neutrophil and central memory CD8 T cell infiltration, suggesting that TRIB1 may play a key role in regulating neutrophil activity and quantity as well as maintaining CD8 T cell stability. XBP1 is an important transcription factor involved in endoplasmic reticulum stress, protein folding and degradation, and the regulation of immune cell functions (42–44). In this study, XBP1 was downregulated in AS patients and negatively linked to neutrophil infiltration, suggesting that it may inhibit excessive neutrophil response under specific conditions, thereby alleviating inflammation.

AS patients often exhibit iron metabolism disorders, such as anemia and iron overload, accompanied by enhanced oxidative stress and elevated lipid peroxidation levels, which are hallmark features of ferroptosis (16, 45). The reduction in antioxidants, including glutathione (GSH) and antioxidant vitamins, observed in patients with AS mirrors the inactivation of the glutathione-dependent enzyme system in ferroptosis (46). This study found that the upregulation of the iron metabolism-related gene SLC40A1 and the lipid metabolism-related gene ACSL1 suggests that ferroptosis may play a critical role in the pathogenesis of AS. The upregulation of SLC40A1 may lead to intracellular iron accumulation, further exacerbating lipid peroxidation and activating inflammatory signaling pathways (37). Similarly, ACSL1 promotes ferroptosis by enhancing lipid oxidation, which significantly correlates with inflammatory markers such as hsCRP identified in this study (35, 47). Additionally, the downregulation of antioxidant-related genes GZMM and XBP1 indicates decreased antioxidant capacity in AS patients, accelerating lipid peroxide accumulation and driving ferroptosis (44).In summary, this study reveals that ferroptosis may contribute to the pathology of AS through disruptions in iron metabolism, enhanced lipid peroxidation, and impaired antioxidant systems.

Additionally, AS is not solely a chronic inflammatory disorder, but also displays features associated with autoimmune pathology (2). Research has indicated that neutrophils would accumulate at inflammation sites in AS, releasing various cytokines and chemokines that drive inflammation progression (48–50). Additionally, the proportion of activated CD8 T cells and dendritic cells is reduced in AS patients (7, 51). This study analyzed the infiltration of 28 immune cell subsets in the AS group and the control group, identifying notable discrepancies in immune cell infiltration levels between the two groups, thereby reinforcing the autoimmune attributes of AS. We then examined the relationship between key genes and six significantly different immune cells, as well as the correlation between key genes and peripheral blood neutrophils. The results showed that the increase in neutrophils in AS was positively correlated with these genes. The study also found a decrease in the proportions of activated CD8 T cells, dendritic cells, TH1 cells, and γδT cells in AS patients, indicating that these cells are essential to the immune surveillance function in AS. Additionally, the AS group showed a notable increase in central memory CD8 T cells, which may reflect a sustained immune response and the establishment of memory cells. These findings provide new clues for further understanding the pathological mechanisms of AS.

The key genes identified in this study (ACSL1, SLC40A1, GZMM, TRIB1, and XBP1) demonstrated favorable diagnostic performance and were associated with disease activity in AS patients. These biomarkers hold considerable potential for clinical applications. For instance, their expression levels could serve as sensitive indicators for early diagnosis, facilitating the identification of high-risk individuals prior to symptom onset. Additionally, dynamic monitoring of these genes’ expression levels may help evaluate disease activity and therapeutic responses, providing valuable guidance for personalized treatment strategies. Notably, genes associated with ferroptosis and immune infiltration, such as ACSL1 and SLC40A1, may serve as promising targets for future therapeutic interventions. Future research should focus on validating these biomarkers in larger, multi-center cohorts and standardizing their use in clinical practice to develop effective diagnostic tools or therapeutic approaches. Furthermore, these biomarkers may achieve greater predictive performance when combined with other clinical parameters, such as imaging or traditional inflammatory markers, thereby improving the accuracy of AS diagnosis and comprehensive disease management.

Although this study provides new insights into the molecular mechanisms of AS, it has several limitations. First, the relatively small sample size may limit the generalizability of the results. Future research should address this limitation by replicating these results in larger, multi-center. Second, this study primarily relies on gene expression data from PBMCs, which may not fully capture pathological changes in other relevant tissues or cell types. Moreover, while gene functions were inferred through bioinformatics methods, experimental validation is required further to enhance the reliability and applicability of the study results.

## 5 Conclusion

The core genes identified in our study demonstrated high diagnostic performance in distinguishing AS patients from healthy individuals, and their expression levels were associated with PBMCs and disease activity. Additionally, this study disclosed a potential association between AS and ferroptosis. Also, multiple core genes and key pathways related to AS pathogenesis were identified through comprehensive analysis. These core genes may serve as potential biomarkers and targets for the diagnosis and treatment of AS. The findings of our study offered new insights that will enable a better understanding of the molecular mechanisms underlying AS and the development of innovative diagnostic and therapeutic strategies.

## Data Availability

The original contributions presented in the study are included in the article/supplementary material. Further inquiries can be directed to the corresponding authors.
